# Gestational diabetes mellitus and polycystic ovary syndrome, a position statement from EGOI-PCOS

**DOI:** 10.3389/fendo.2025.1501110

**Published:** 2025-01-31

**Authors:** Paola Quaresima, Samuel H. Myers, Basilio Pintaudi, Rosario D’Anna, Michele Morelli, Vittorio Unfer

**Affiliations:** ^1^ Department of Obstetrics and Gynecology, Azienda Sanitaria Provinciale di Cosenza, Cosenza, Italy; ^2^ R&D Department, Lo.Li Pharma s.r.l, Rome, Italy; ^3^ The Experts Group on Inositol in Basic and Clinical Research and on PCOS (EGOI-PCOS), Rome, Italy; ^4^ Azienda Socio Sanitaria Territoriale (ASST) Grande Ospedale Metropolitano Niguarda, Milan, Italy; ^5^ Department of Human Pathology, University of Messina, Messina, Italy; ^6^ Department of Obstetrics and Gynecology, Annunziata Hospital, Cosenza, Italy; ^7^ UniCamillus-Saint Camillus International University of Health Sciences, Rome, Italy

**Keywords:** gestational diabetes mellitus, polycystic ovary syndrome, insulin, metformin, myo-inositol

## Abstract

Gestational diabetes mellitus is a worldwide health issue in pregnancy, posing a threat to both mother and child. One of the major risk factors for the development of gestational diabetes mellitus is polycystic ovary syndrome, primarily due to the biochemical hyperandrogenism and metabolic issues, commonly observed in these patients. In recent years, the Expert Group on Inositol in Basic and Clinical Research and on PCOS (EGOI-PCOS) has sought to better understand the pathogenesis behind polycystic ovary syndrome, in order to accurately diagnose and treat patients according to their individual needs. Through the scope of polycystic ovary syndrome, this position paper examines the characteristics of both conditions, and underlying biological mechanisms, before moving on to common treatment strategies to avoid or treat gestational diabetes mellitus in women with polycystic ovary syndrome.

## 1 Introduction

Gestational diabetes mellitus (GDM), is currently the most common medical complication of pregnancy (1). The reported GDM prevalence varies substantially worldwide, ranging from 7.1% in North America and the Caribbean to 27.6% in North Africa and the Middle East, with a lack of uniformity in the screening standards and diagnostic criteria, presenting a challenge for prevalence studies (2). GDM poses a health risk to pregnant women as it is associated with adverse pregnancy outcomes such as pre-eclampsia, polyhydramnios, shoulder dystocia, fetal macrosomia, neonatal hypoglycemia, and in extreme cases, perinatal mortality (1). The actual GDM screening strategies varies across the world, from a universal screening approach, to selected population screens targeted at those women considered to be at risk for GDM, such as women with PCOS. Both conditions are associated with metabolic alterations, with insulin resistance being observed in a majority of PCOS patients. This shared clinical feature, predisposes women with PCOS to develop GDM during pregnancy.

Polycystic ovary syndrome (PCOS) is the most common endocrine disorder in females of reproductive age, affecting 10-13% of women worldwide (3, 4). As PCOS can present with a varied range of clinical symptoms, it is important to have clear criteria and advice for patients and physicians alike to help guide diagnosis and treatment. Consequently, several diagnostic criteria have been published regarding the identification of PCOS, for the past 20 years, the most applied of these has been the Rotterdam Criteria. These criteria describe PCOS as a condition with at least two out of three of the following: biochemical and/or clinical hyperandrogenism, oligo/anovulation, and polycystic ovarian morphology (5), consequently dividing the patient cohort into four phenotypes:

- Phenotype A: biochemical and/or clinical hyperandrogenism, oligo/anovulation, and polycystic ovarian morphology.
- Phenotype B: biochemical and/or clinical hyperandrogenism, oligo/anovulation.
- Phenotype C: biochemical and/or clinical hyperandrogenism, polycystic ovarian morphology.
- Phenotype D: oligo/anovulation, and polycystic ovarian morphology.

Of note, three of these phenotypes contain hyperandrogenism, with one phenotype (D) being non-hyperandrogenic. This difference has been a point of contention with numerous societies such as the AE-PCOS and the EGOI-PCOS arguing that the non-hyperandrogenic subgroup represents a separate condition (6, 7). Women with PCOS typically suffer from insulin resistance in addition to other metabolic issues; however, it is notable that this is more commonly observed in hyperandrogenic PCOS patients. Consequently, it may be argued that these metabolic alterations in these patients are a key part of the pathogenesis of the condition (8). Despite insulin resistance’s crucial role in PCOS, it was not included as a diagnostic in the most recent iteration of the international clinical guidelines (9). In 2023, the EGOI-PCOS laid out the EGOI-PCOS criteria, which reclassified hyperandrogenic PCOS as endocrine-metabolic syndrome (EMS), including insulin resistance as a fundamental part of the diagnosis. For the patients with phenotype D, the name multi-follicular ovarian syndrome (MFOD) was proposed, with these patients being formally separated from EMS due to them not presenting with hyperandrogenism nor insulin resistance (10).

Considering the significant association between GDM and PCOS, this article describes the link between these conditions and how this differs between patient groups, in order to discuss therapeutic approaches that can be utilized in the treatment of both conditions, with the aim of aiding patient care.

## 2 Prevalence

An association between PCOS and GDM has been established in the literature with PCOS thought to be a major causal factor for GDM (11). In detail, GDM develops in approximately 40% of pregnancies in women with PCOS (12). This is evidenced in a recent meta-analysis Qiu et al. demonstrated that PCOS was associated with an increased risk of GDM via a random-effects model (OR 2.02, 95% CI: 1.74–2.34, p < 0.0001) (13). In a further meta-analysis of 33 studies and a sample size of 92,819, PCOS was significantly associated with an increased risk of GDM (OR 1.51, 95% CI:1.17–1.94) (14). Interestingly, the prevalence of GDM changes depending on the presented PCOS phenotype, those characterized by the presence of hyperandrogenism and oligomenorrhea, such as A and B, and C (EMS) were at a significantly increased risk of developing diabetes during pregnancy in comparison to phenotype D (or MFOD according to EGOI-PCOS) (15). Furthermore, in a multicenter cohort study maternal complications were significantly higher in women with hyperandrogenic PCOS (adjusted OR 2.67, 3.47-8.87) versus their normoandrogenic counterparts (1.60, 0.74-3.49) (16).

Accordingly, the most recent guidelines an OGTT should be performed in all women without pre-existing diabetes, when planning pregnancy or seeking fertility treatment due to the high risk of hyperglycemia and associated comorbidities during pregnancy (9).

As previously stated, the EGOI-PCOS has proposed a formal reclassification of PCOS, with a separation of typically insulin resistant-hyperandrogenic PCOS patients, and typically normoandrogenic PCOS patients who do not typically demonstrate insulin dysfunction. The redivision of these patient groups aims to improve patient care, as current therapeutic approaches, namely the use of oral contraceptives and insulin sensitizers, are not applicable to the normoandrogenic cohort. Consequently, normoandrogenic PCOS patients lack suitable therapeutic options, and it is hoped that the EGOI-PCOS reclassification will encourage further research into appropriate treatments for this patient group. As it pertains to GDM, women with phenotype D (or MFOD according to EGOI-PCOS classification) have a reduced risk of GDM and thus pregnancy advice regarding GDM should be tailored accordingly.

## 3 Risk factors and screening methods for GDM

Women with PCOS are characterized by multiple well-known risk factors for GDM, with the most common of these risk factors being elevated BMI (33-88% of women with PCOS BMI >25 kg/m^2^) (17). Irregular menstrual patterns are also an independent risk factor for the development of GDM (18). While the specific rationale for this observation is not well understood, it is hypothesized that irregular menstrual cycles could be an indicator of a hormonal and metabolic imbalance, which is thought to play a role in GDM (19). Other associated risk factors for GDM include an elevated free androgen index (FAI), as SHBG is negatively associated with further risk of GDM (20).

Familial history of type 2 diabetes represents a significant risk factor for the development of GDM, as evidenced by a cohort study including 1129 pregnant women with first- or second-degree relatives with T2DM. Women with first-degree relatives and/or women with second-degree relatives with T2DM demonstrated a significant higher prevalence of GDM (26.6%, 26.3%, and 33.3% respectively) versus negative controls (15.9%) (21).

Insulin resistance, an integral part of GDM, is associated with other common endocrine disorders such as thyroid dysfunction. A retrospective study of 662 pregnant women, 412 of which had GDM, demonstrated that women with GDM had a significantly higher concentration of TSH, in addition to a high FT3:FT4 ratio (22). Consequently, these biomarkers may have potential in the future to identify increased risk of GDM in patients seeking pregnancy. A similar relationship is observed in PCOS patients where that the incidence of hypothyroidism is higher (11–14%) compared with healthy controls (1–2%) (23). Moreover, GDM, hypothyroidism and PCOS share common metabolic symptoms or comorbidities including insulin resistance, dyslipidemia, and obesity, which may explain the correlation between PCOS, thyroid dysfunction, and GDM incidence.

Numerous societies and organizations have attempted to standardize diagnosis and screening methods to identify GDM. The World Health Organization (WHO) defines GDM as “any level of the early of first detection of glucose intolerance in pregnancy (24), and set out diagnostic and screening criteria in 1991, recommending the use of an 2h 75g OGTT as the standard diagnostic test with a fasting and 2h threshold of 126 and 140 mg/dl respectively (25). The OGTT test typically is performed between the 24^th^ and 28^th^ week of pregnancy. Alongside the WHO criteria, the International Association of Diabetes in Pregnancy Study Groups (IADPSG) criteria represent the most globally applied diagnostic criteria (26). Much like the WHO criteria, the IADPSG recommend the use of a 2h 75g OGTT but with the following cutoffs (Fasting glucose: 95 mg/dl, 1h 180 mg/dl, 2h 153 mg/dl). In 2013 the WHO updated the 1999 guidelines bringing them into line with the IADPSG recommendations (27). It should be noted that the IADPSG criteria are not routinely used in the United States and Canada, as the American Diabetes Association recommends the use of a 100g 3h OGTT (28). Additionally, the UK does not follow the IADPSG guidelines, instead following National Institute for Health and Care Excellence (NICE) guidelines, which recommend the use of a 2h OGTT (Fasting glucose 101 mg/dl, 2h 140mg/dl). The NICE guidelines differ from the recommendations from other societies through the advocation for selective risk-factor-based testing (29). Risk factors considered by the NICE guidelines include BMI >30, prior macrosomic baby weight of >4.5kg, previous incidence of GDM, family history of diabetes, an ethnicity based factors (30). The diagnostic and screening criteria are summarized in **Table 1**.

**Table 1:** Summary of diagnostic and screening criteria for GDM- adapted from Choudhury et al. (26).

Criteria	Glucose (g)	Time (h)	Glucose threshold (mg/dl)				Ref
			Fasting	1 h	2 h	3 h	
WHO (1999)	75	2	126	–	140	–	(25)
ADA (2004)	100	3	95	180	155	140	(28)
IADPSG (2010)	75	2	92	180	153	–	(26)
WHO (2013)	75	2	95	180	153	–	(27)
NICE (2015)	75	2	101	–	140	–	(30)

As PCOS increases the risk of development of GDM, screening protocols must reflect the specific needs of these women. The 2023 International Evidence-based Guideline for the Assessment and Management of Polycystic Ovary Syndrome advised an OGTT in women with PCOS and without pre-existing diabetes prior to planning pregnancy or seeking fertility treatment (31). If this OGTT is not performed, an OGTT should be offered at the first prenatal visit, in addition to another test between the 24^th^ and 28^th^ week of gestation. This advice is in line with the current recommendation of EGOI-PCOS. To the best of our knowledge, the benefits of early screening in PCOS populations have not been studied; however, some conclusions may be drawn from studies investigating the effect of early screening in GDM patients. In detail, hyperglycemia at the first prenatal visit is a known predictor of GDM as observed in a study by Zhu et al. which underlined the importance of measuring fasting plasma glucose at this visit (32). Furthermore, a retrospective cohort study conducted by Liu et al, demonstrated that while the measurement of fasting plasma glucose at the first prenatal visit was not associated with lipid concentrations in mid-pregnancy, it was associated with numerous fetal outcomes such as birthweight, head circumference, and shoulder circumference (33). Considering the prevalence of hyperglycemia in PCOS, the above findings support the implementation of early screening protocols in PCOS patients.

## 4 Insulin signaling in GDM and PCOS

PCOS is commonly associated with insulin resistance (IR), a term used to describe a decrease in the cellular response to insulin signaling which subsequently induces an increase in insulin secretion (34). During insulin resistance in PCOS, higher insulin levels lead to the reduced liver synthesis and reduced secretion of SHBG. A reduction of SHBG levels results in higher bioavailable testosterone, which is associated with the impairment islets of Langerhans function, thereby impairing pancreatic function which leads to further IR (35). Consequently, increased systemic insulin levels result in an increase in insulin-dependent androgen overproduction within ovarian theca cells, thereby creating a vicious circle where IR leads to hyperandrogenism and vice versa (36).

In order to facilitate proper growth of the fetus, correct nutrient flow across the placenta must be regulated during pregnancy. Transport of glucose across the placenta is performed by facilitated transport facilitated by glucose transporters (GLUTs) and is dependent upon fetal and maternal systemic concentrations (37). In the later stages of pregnancy, the fetal need for glucose increases, thus glucose transport is increased, which has the potential to lower maternal glucose levels. Consequently, maternal physiological endocrine changes occur, increasing maternal insulin resistance and hepatic glucose production (38). However, this process may result in the development of GDM in cases where pancreatic β-cell function is insufficient to overcome not only the physiological IR associated with pregnancy, but IR caused by metabolic risk factors such as those commonly presented in PCOS (39).

From a molecular viewpoint, GDM acts primarily on three different tissues: placenta, adipose, and skeletal muscle (40). Within the placenta, GDM induces an increase in mTOR, NFKB, and TLKR3 signaling, in addition to endoplasmic reticulum (ER) stress; meanwhile, PI3K, SIRT, AMPK and PPAR signaling is reduced. Within the adipose tissue GSK3, NOD, PI3K, and ER stress are increased, while AMPK and PPAR signaling are decreased. Lastly within skeletal muscle AMPK, PI3K, and SIRT signaling are decreased, while ER stress and GSK3 signaling are increased. The net result of these molecular alterations are placental and endothelial disruption, increased inflammation and an increased level of insulin resistance within the mother, which can go on to cause long and short-term effects within the offspring. Endothelial disruption, inflammation, and insulin resistance are hallmarks of PCOS, which can worsen GDM, providing an explanation for why PCOS patients are at risk of developing GDM.

## 5 Maternal fetal complications of GDM

The characteristic PCOS features, obesity, IR, and hyperandrogenism have significant implications for both short and long-term pregnancy outcomes. Evidence suggests that women with PCOS may be at greater risk for various obstetric complications, aside from GDM, such as hypertensive disorders of pregnancy, premature birth, induction of labor and cesarean delivery (41, 42). In detail, a meta-metanalysis of 63 studies observed that women with PCOS were more likely to have gestational hypertension (29 studies, OR: 2.58, 95% CI: 1.95‐3.41), preeclampsia (26 studies, OR: 1.87, 95% CI: 1.55‐2.25), induction of labor (5 studies, OR: 1.87, 95% CI: 1.55‐2.25), and caesarean section (25 studies, OR: 1.37, 95% CI: 1.21‐1.56) (11). Moreover, the presence of PCOS together with GDM leads to a 2.4-fold increase in risk of hypertension and a 2-fold increase in risk of preeclampsia compared to GDM alone (43). Furthermore, in a retrospective population study over a period of 11 years conducted by Mills et al, an increased risk of GDM was observed women with PCOS vs the control group (aOR 2.19, 95% CI 2.02–2.37), and women within the PCOS group were also more likely to develop chronic hypertension (aOR 1.38, 95% CI 1.27–1.50, P < 0.001), gestational hypertension (aOR 1.47, 95% CI 1.31–1.64), and preeclampsia (aOR 1.29, 95% CI 1.14–1.45) (44).

GDM and PCOS pose significant risks to the fetus in addition to the mother. This was apparent in a case-controlled study that demonstrated that neonatal hypoglycemia risk is increased in patients with both GDM and PCOS compared to those with GDM alone, with an incidence of 17% and 5% respectively between the two groups (43). Moreover, among women with PCOS, the odds of developing neonatal jaundice and respiratory complications were significantly higher compared to the non-PCOS control group (45). Of note, while the majority of evidence from the literature indicates that neonates of mothers with GDM and PCOS were more likely to be of a lower birthweight compared to neonates from mothers with GDM alone, others described opposite results, with higher birthweight and macrosomia (46).

In addition, women with GDM have an increased risk of developing metabolic syndrome, notably, this effect is also observed in offspring of women with GDM compared to offspring not exposed to GDM *in utero* (47). This risk is further compounded by the increased prevalence metabolic syndrome observed in PCOS patients (48).

Considering these increased risks to both mother and child, medical care must be tailored to suit the individual needs of these women. As lifestyle changes represent the first recommendation in both conditions, appropriate diet and exercise counseling should be made available to the patient, with diet and exercise plans in addition to frequent follow-ups sessions to ensure compliance. Moreover, as pregnancies in women with GDM and/or PCOS are associated with higher degree of pregnancy complications, psychological support should be made available. A systematic review of 44 studies, observed that women with GDM had increased rates of anxiety or depression during pregnancy. Furthermore, the presence of anxiety or depression during pregnancy increased the incidence of GDM suggesting a cyclic effect (49). As PCOS is also associated with mental health issues (50), appropriate psychological intervention in these women may lower GDM incidence or reduce the impact of GDM in these women. Lastly women with PCOS should be informed of the risk of developing GDM when planning contraception or undergoing fertility care.

## 6 Treatment strategies for GDM

### 6.1 Lifestyle changes

The primary goal of GDM treatment is the prevention of excessive fetal growth and GDM related pregnancy complications. According to the “Pedersen hypothesis” increased maternal glycaemia directly associates with both excessive fetal growth and other unwanted complications associated with GDM (51). Consequently, the primary goal of GDM management and treatment is to achieve glycemic control, therefore lifestyle modifications are routinely initiated following diagnosis (52). It is recommended that pregnant women with or at risk of GDM consume a hypoglycemic diet including three main meals and 2–4 snacks, comprising of ≥175 g of carbohydrates daily, in order to insure appropriate fetal growth and cerebral development (53). Exercise also represents a core component of lifestyle modification, aerobic and resistance exercise at a moderate intensity, a minimum of three times a week for 30-60 min is recommended for women with/or at risk of GDM (54). In total, lifestyle changes have been demonstrated to enable up to 80% women to reach their glycemic targets (52).

### 6.2 Insulin therapy

While lifestyle changes are sufficient for the majority of patients, insulin therapy represents a valid approach when glycemic control cannot be established. Despite this, no clear indications exist to guide when a patient should begin insulin therapy. Typically, insulin therapy is typically initiated when glucose levels are between 5.2 and 5.6 mmol/L during fasting and 6.6-7.9mmol/L during at the second or third trimester of pregnancy (17). The basal insulins approved for use in pregnancy are the neutral protamine Hagedorn (NPH), an intermediate-acting insulin, and the long-acting insulin analog detemir (55). The short-acting insulin analogs aspart, lispro, and the long-acting detemir are approved by the U.S. Food and Drug Administration (FDA) as category B drugs and are commonly used in pregnancy (56), and have been demonstrated to mirror the endogenous pattern of insulin secretion. The long-acting insulin analogs glargine and degludec have not received official authorization for use in pregnancy; however, several observational studies exploring the use of insulin glargine in pregnancy report a similar safety profile compared to NPH insulin (57). In addition, in patients who are accustomed to the use of glucose monitoring devices continuous rapid acting insulin analogues may be employed, typically in women who have progressed to GDM or suffer from type-1 diabetes mellitus (58). Clinical benefits from insulin therapy include reduced incidence of macrosomia, lower cranial thoracic circumference ratio and a reduced incidence of caesarean sections. Benefits of insulin therapy depend on the type of insulin used with long-acting insulin resulting in a higher incidence of macrosomia vs short acting insulin treatments (59). It is important to consider the associated risks when prescribing insulin therapy, which include of hypertensive disorder in pregnancy, gestational weight gain, labor induction, impaired placental insulin signaling, and reduced birthweight (58).

### 6.3 Metformin

Oral glucose lowering medications, such as metformin, have been studied in women with GDM due to their demonstrated worth in related diseases such as type 2 diabetes (60). Metformin primarily functions by suppressing hepatic glucose production, leading to a reduction in fasting plasma glucose levels (61). Metformin is routinely prescribed in women with PCOS who suffer from metabolic abnormalities. In a recent meta-analysis of 32 RCTs, use of metformin in patients with PCOS resulted in significant reductions to BMI, HOMA, and fasting glucose levels, with a moderate certainty of evidence (62). However, these metabolic changes were coupled with a significant increase in mild gastrointestinal adverse effects compared to the placebo. Regarding hormonal changes, in the same meta-analysis total testosterone was reduced in the metformin group versus placebo, albeit with a very low certainty.

Concerning metformin use during pregnancy, according to a recent meta-analysis, metformin administration showed mixed results in pregnant women with PCOS as it was associated with a reduced preterm delivery risk; however, metformin use was also associated with a larger neonatal head circumference (63). Moreover, a large population-based cohort study revealed that metformin supplementation in women with PCOS reduced the risks preeclampsia, gestational diabetes, cesarean section, and preterm birth in comparison to the control PCOS group; however, metformin supplementation in women without PCOS may increase the risk of obesity in offspring (64). Furthermore, metformin is associated with gastrointestinal adverse effects which may not be tolerable for some patients (65). It should be noted that metformin crosses the placenta, and maternal and fetal concentrations have been demonstrated to be comparable; however, no evidence of teratogenicity nor short term adverse neonatal outcomes has been reported, while long term affects are still unclear (66).

The use of metformin and insulin was compared by Wu et al. in a meta-analysis which compared 24 randomized controlled trials (RCTs) involving 4934 patients with GDM. Compared to insulin, metformin demonstrated a significantly reduced risk of preeclampsia, induction of labor, cesarean delivery, macrosomia, neonatal intensive care unit admission, neonatal hypoglycemia, and large for gestational age (67). Despite the promising results of metformin vs insulin in GDM, further study is required to measure the effect of GDM in PCOS patients in comparison to insulin therapy (68). A recent study on two open label RCTs investigated the benefits of metformin vs insulin in terms of post birth outcomes. The offspring from the original cohorts of the prior studies were followed up after 9 years, following this period no difference was observed in terms of offspring growth and glucose metabolism; however, the lipid profile in the metformin group was significantly improved over the insulin cohort (69). A recent metanalysis investigated the effect of metformin and insulin treatment during pregnancy on the cardiometabolic outcomes in offspring. This meta-analysis conducted by Rawat et al. consisted of five RCT studies (metformin 409 children, insulin 434 children). The metformin group demonstrated significantly higher fat free mass, and lower triglyceride and plasma glucose levels between the ages of 5 and 9 than the insulin group (70). Aside from these observations, no other cardiometabolic differences could be identified between the two groups. In cases where glycemic control cannot be achieved, patients with GDM taking metformin are recommended insulin therapy, occurring in approximately a third of patients (71).

In terms of its tolerability, metformin is generally regarded to have an acceptable safety profile. The most common adverse effect of metformin treatment is gastrointestinal effects, which may render metformin intolerable for some patient groups (65). Metformin is contraindicated in patients with severe renal impairment, due to its association with lactic acidosis which may occur in a minority of patients. Due to this concern metformin has received a black-box warning from the FDA regarding lactic acidosis (72). Consequently, these safety concerns should be considered when prescribing metformin in pregnant individuals.

### 6.4 Myo-inositol

Inositol is a naturally occurring polyol which is present in numerous food sources including cereals, legumes, seeds, and nuts (73). In nature the two most common stereoisomers of inositol are myo-inositol (MI) and D-chiro-inositol, these molecules function as second messengers of various endocrine signals including follicle stimulating hormone (FSH), thyroid stimulating hormone (TSH) and insulin (74). Under insulin stimulation, MI is converted to DCI via a unidirectional tissue-specific epimerase, maintaining a balance between DCI and MI, ensuring normal metabolic function (74). Inositol is involved in various steps of the insulin signaling pathway and can act as an insulin sensitizer (75). In detail, MI ameliorates IR by encouraging the translocation of GLUT4 to the plasma membrane to increase glucose uptake. Similar to the action of metformin, this function of MI against IR reduces insulin-dependent androgen signaling and can counteract hyperandrogenism in women with PCOS (76). Moreover, MI demonstrates non-inferiority against metformin in treating PCOS across most measurable outcomes. In a recent meta-analysis of 26 RCTs by Greff et al., endocrine-metabolic outcomes such as changes to BMI, free testosterone, total testosterone, androstenedione, and glucose levels in addition to AUC were equivalent between MI and metformin (77). Moreover, in the same analysis MI supplementation was associated with a normal menstrual cycle and increased sex hormone binding globulin level (SHBG) levels versus placebo.

Numerous studies have evaluated the use of MI in PCOS patients at risk with or who have developed GDM. A retrospective study in pregnant women with PCOS demonstrated that MI supplementation halved the risk of GDM in comparison to the control group (78). Moreover, in the same study MI was well tolerated throughout pregnancy with no adverse effects reported for the fetus or the mother. A recent meta-analysis evaluated patients undergoing MI supplementation in comparison to routine treatment across four RCTs. In total, MI supplementation lead to a significantly reduced requirement for insulin treatment and HOMA; however, no difference was observed in birth weight, incidence of cesarean section or the requirement for administration to the neonatal intensive care unit (79). An earlier meta-analysis compared the results of 7 RCTs investigating the use of inositol (either MI or a combination of MI/DCI) in the prevention of GDM (73). In the analyzed trials, use of 4g of MI/day reduced the incidence of GDM, plasma glucose levels as measured by OGTT and 2h OGTT. Furthermore, MI supplementation reduced several secondary outcomes such as the need for insulin treatment and reduced the incidence of preterm delivery and neonatal hypoglycemia. It should be noted that the combination of MI (1.1g) and DCI (27.6 mg) was equivalent to the control group across all measured parameters, suggesting the use of MI alone is more effective and may represent a potential therapeutic in the GDM disease space. Further studies are required to fully evaluate the use of MI in GDM. In a meta-analysis of six RCTs including 887 women, MI supplementation was observed to potentially reduce the risk of GDM (RR 0.54; CI [0.30, 0.96]); however, the certainty of evidence was classified as low to very low (80). Furthermore, the same meta-analysis identified no adverse outcomes due to MI supplementation, and a reduction of preterm delivery and pregnancy induced hypertension; however, once more the strength of evidence was classified as low to very low. Furthermore, to the best of our knowledge no meta-analyses have addressed the use of myo-inositol in patients with both PCOS and GDM demonstrating an urgent need for further study.

In terms of its safety profile, MI is well-tolerated and is included in the list of generally recognized as safe (GRAS) food additives. Gastrointestinal effects are only observed in very high doses of MI (over 12g/day), which are excessive of what is typically recommended (81). Considering these factors MI does not have any contraindications.

### 6.5 Treatment options in GDM vs GDM and PCOS

As noted above, treatment options for GDM primarily consist of lifestyle adjustments, in addition to insulin therapy when required. Furthermore, insulin sensitizers such as metformin and inositol, have potential in reducing the need or dose of insulin therapy; however, the long-term implications of metformin are yet to be discovered. Once pregnancy has been achieved this treatment plan is also recommended for patients with both GDM and PCOS. Prior to conception PCOS patients may be recommended therapies aimed at treating hyperandrogenism and restoring menstrual cyclicity. Clomiphene and more recently letrozole are routinely employed to induce ovulation; however, once pregnancy is achieved this therapy is stopped (82). Patients with PCOS frequently develop signs of clinical hyperandrogenism such as hirsutism, acne, and alopecia. Cosmetic treatments such as laser hair removal can be continued during pregnancy, as may topical anti-androgenic creams with the exception of hydroquinone and tretinoin which may have increased systematic absorption rates (83).

## 7 Conclusion

Women with PCOS are a higher risk of developing gestational diabetes during pregnancy. It is, however, important to contextualize this potential risk, as hyperandrogenic PCOS phenotypes are known to be a higher risk group than normoandrogenic PCOS patients. The characteristic hormonal and metabolic profile of hyperandrogenic PCOS patients includes insulin resistance, and an elevated FAI, both of which are risk factors for the development of GDM. Women with PCOS are at further risk of other obstetric complications including hypertensive disorders, premature birth, induction of labor and cesarean delivery.

## 8 Key findings

Due to the elevated risk of developing GDM, an OGTT is advised in all women with PCOS and without pre-existing diabetes, when planning pregnancy or seeking fertility treatment. If not performed prior to conception, an OGTT may be performed at the first antenatal visit or at least prior to 24-28 weeks gestation. In the incidence of a GDM diagnosis, healthy diet and physical activity should be promptly recommended, and if glycemic targets are not reached insulin treatment should be considered as a first line therapy. In addition, pharmacological insulin sensitizers such as metformin may have merit; however, potential concerns remain regarding long term adverse effects in the offspring. As an alternative to metformin, MI has shown potential for GDM prevention in PCOS populations.

## 9 Research questions

The connection between PCOS and pregnancy complications, such as GDM, have been well described within the literature. Described below are some of the key research questions that merit further research according to the EGOI-PCOS:

- As a society, the EGOI-PCOS are seeking to reclassify PCOS into EMS and MFOD; however, it is yet to be known whether this reclassification would affect the association with GDM between both groups.
- Is there a difference in how the subgroups of PCOS (i.e. hyperandrogenic vs non-hyperandrogenic) respond to therapies designed to treat GDM.
- Further larger powered studies are required to evaluate the efficacy of insulin sensitizers in GDM treatments. Furthermore, the long-term effects of metformin in offspring should be studied.
- The benefits of early screening for GDM in PCOS patients in terms of pregnancy outcomes should be investigated further.

## Data Availability

The original contributions presented in the study are included in the article/supplementary material. Further inquiries can be directed to the corresponding author.
