# Corrigendum: Gestational diabetes mellitus and polycystic ovary syndrome, a position statement from EGOI-PCOS

**DOI:** 10.3389/fendo.2025.1569086

**Published:** 2025-02-26

**Authors:** Paola Quaresima, Samuel H. Myers, Basilio Pintaudi, Rosario D’Anna, Michele Morelli, Vittorio Unfer

**Affiliations:** ^1^ Department of Obstetrics and Gynecology, Azienda Sanitaria Provinciale di Cosenza, Cosenza, Italy; ^2^ R&D Department, Lo.Li Pharma s.r.l, Rome, Italy; ^3^ The Experts Group on Inositol in Basic and Clinical Research and on PCOS (EGOI-PCOS), Rome, Italy; ^4^ Azienda Socio Sanitaria Territoriale (ASST) Grande Ospedale Metropolitano Niguarda, Milan, Italy; ^5^ Department of Human Pathology, University of Messina, Messina, Italy; ^6^ Department of Obstetrics and Gynecology, Annunziata Hospital, Cosenza, Italy; ^7^ UniCamillus-Saint Camillus International University of Health Sciences, Rome, Italy

**Keywords:** gestational diabetes mellitus, polycystic ovary syndrome, insulin, metformin, myo-inositol

In the published article, there was an error in **Table 1** as published. The fasting glucose for IADPSG cut off was written as 95, this was a typo, as it should be 92. The correct table appears below.

**Table 1:** Summary of diagnostic and screening criteria for GDM- adapted from Choudhury et al. (26).

Criteria	Glucose (g)	Time (h)	Glucose threshold (mg/dl)				Ref
			Fasting	1h	2 h	3 h	
WHO (1999)	75	2	126	–	140	–	(25)
ADA (2004)	100	3	95	180	155	140	(28)
IADPSG (2010)	75	2	92	180	153	–	(26)
WHO (2013)	75	2	95	180	153	–	(27)
NICE (2015)	75	2	101	–	140	–	(30)

In the published article, there was a typo in the text.

A correction has been made to *Section 6.4*, Paragraph 1. This sentence previously stated:

“In nature the two most common stereoisomers of inositol are myo-inositol (MI) and D-chiro-inositol, these molecules function as second messengers of various endocrine signals including follicle stimulating hormone (FSH), thyroid stimulating hormone (LH) and insulin (74).”

The corrected sentence appears below:

“In nature the two most common stereoisomers of inositol are myo-inositol (MI) and D-chiro-inositol, these molecules function as second messengers of various endocrine signals including follicle stimulating hormone (FSH), thyroid stimulating hormone (TSH) and insulin (74).”

The authors apologize for this error and state that this does not change the scientific conclusions of the article in any way. The original article has been updated.

